# *C*^1^ triangular Coons surface construction and image interpolation based on new Side-Side and Side-Vertex interpolation operators

**DOI:** 10.1371/journal.pone.0231617

**Published:** 2020-04-22

**Authors:** Mingshan Qiu, Yuanpeng Zhu

**Affiliations:** School of Mathematics, South China University of Technology, Guangzhou, China; Consiglio Nazionale delle Ricerche, ITALY

## Abstract

In this paper, a new class of rational quadratic/linear trigonometric Hermite functions with two shape parameters is proposed. Based on these Hermite functions, new improved first class of Side-Side (FCSS), second class of Side-Side (SCSS), first class of Side-Vertex (FCSV) and second class of Side-Vertex (SCSV) interpolation operators are proposed respectively, which can be used to construct *C*^1^ Coons surfaces over triangular domain. By altering the values of two shape parameters, the shape of the Coons surface patch can be adjusted flexibly, but without affecting the function values and partial derivatives of the boundaries. For constructing the triangular surface patches with the center of mass passing through a fixed point, we also give a center of mass function value control method, by which we can solve the corresponding shape parameter values. Moreover, we also apply these four improved interpolation operators to image interpolation. Compared with some widely used image interpolation methods, our methods achieve competitive performance.

## 1.Introduction

The problem of constructing surfaces through a given set of scattered data points has a wide range of applications in many fields, such as medical imaging, scientific visualization, architectural design and aerospace materials manufacturing. In order to satisfy various requirements and obtain pleasing surfaces, many methods have been proposed, such as NURBS, Bézier-like surfaces, and so on. These methods can accurately construct quadrangular surfaces and free form surfaces. However, they still have disadvantages in some applications. One of them is that when adjusting the shape inside the surface, the boundary function values or partial derivatives will be affected simultaneously, which cannot satisfy the requirement of maintaining the shape of the boundaries.

In order to solve this problem, Coons [1] proposed a new surface construction method. If we need to construct a quadrangular surface that satisfies the boundary interpolation requirement, we only need to provide the data points with four boundaries. In real applications, bilinear Coons patches and bicubic Coons patches are widely used. However, the shape of the classic Coons patch is fixed and related to the given data points. Wang et al. [2] made an improvement and used second-order trigonometric blending functions to construct biquadrate trigonometric polynomial Coons surface patches with adjustable parameters. Zou et al. [3] constructed a rational Coons surface over rectangular domain, which inherits the good properties of bicubic Coons patches. In her method, R-Hermit basis functions are applied to the interpolation operators, and the parameters in basis functions are used to adjust the shape of the patch. The rational Coons surface can accurately represent ellipsoidal surface, elliptical cone surface, and elliptic cylinder surface.

In computer-aided design (CAD), surfaces are often defined over rectangular domain, for CAD is originally applied to the design of objects with rectangular structures such as cars and aircraft fuselages. However, as the application range of surface geometric modeling technology becomes wider, and the shape of the design surface becomes more complex, rectangular surfaces and rectangular topology appear some limitations. Many scholars have begun to study the surface patches of non-rectangular topologies, such as the triangular surface patches. One of the methods used to construct Coons surface patches over triangular domain is transfinite interpolation, which constructs surface patches by interpolating the boundary values. The two main methods of transfinite interpolation are Side-Side method and Side-Vertex method. Barnhill et al. [4] first presented the Side-Side method, which uses Boolean sum to derive rational blending interpolation functions for constructing triangular surface patches that interpolate the boundary values and partial derivatives. Their method requires the cross partial derivatives to be compatible. If not, Gregory’s square [5] can be used to solve this problem. Barnhill et al. [6] used the polynomial blending functions to replace the rational blending functions, and proposed a polynomial Boolean sum interpolant. In [7], Side-Vertex method was proposed by Nielson, which is based upon the convex combinations of three interpolation operators consisting of univariate interpolation along lines segments joining a vertex and its opposing side. In [8], Nielson proposed a new method for constructing *G*^1^ surfaces. This new method utilizes the surface normals rather than the partial derivatives on the boundaries. In [9], Hagen generalized the Side-Vertex method using the geometric Hermite operators. The constructed surface patch guarantees to interpolate the function values, derivatives and curvature values of the triangle boundaries. In [10], this result has been further generalized to open and closed *G*^2^ triangular surface patches by Hagen. Zhang et al. showed a new method to construct triangular surface patches in [11], which is the combination of an interior interpolation operator and three Nielson’s Side-Vertex interpolation operators. This method shows better approximation precision than Nielson’s Side-Vertex method.

The Coons surface patches constructed by the above methods have advantages in continuity and interpolation properties. However, once the boundary values are given, designers cannot further adjust the shape of the surface because it is already fixed. In order to construct adjustable curves and surfaces, researchers have proposed some convenient and effective methods, one of them is to apply shape parameters to the basis functions. In [12], Zhang gave four cubic C-Hermite polynomials depending on parameter *α* > 0, and they can exactly reproduce circles and cylinders. In [13], Han presented quadratic trigonometric polynomial curves with a shape parameter, and those curves satisfy *C*^1^ continuity. In [14], Li et al. introduced two kinds of Coons surface patches generated by the hyperbolic polynomial functions and trigonometric polynomial functions respectively. Both two improved schemes achieve adjustable shape through combining the shape parameters in basis functions. In [15], Wu et al. used two classes of trigonometric Hermite polynomial functions to construct the Coons surface patches over triangular domain. By changing the shape parameters, the shape of the Coons surface patch can be adjusted. In [16], Ibraheem et al. developed a scheme for constructing positive surfaces via parameter control.

Interpolation is also an important tool in image magnification and visual processing. Image interpolation is an image processing technique that uses low resolution images (LR) to obtain high resolution images (HR). Nearest-neighbor interpolation, bilinear interpolation and bicubic interpolation are classic image interpolation algorithms. Bicubic interpolation was proposed by Keys in [17], and it shows an order of accuracy between that of linear interpolation and that of cubic spline. An algorithm for image enlargement and reduction with arbitrary accuracy using the scaling function of B-spline wavelet was offered by Yang et al. in [18]. An algorithm of image smooth interpolation using cubic B-spline was proposed by Hou et al. in [19], which can perfectly implement image enlargement, reduction and noise smoothing. In recent years, many new image interpolation algorithms have been proposed. In [20], Takeda et al. applied kernel regression to image interpolation and reconstruction. This algorithm is ideal for processing noisy images and sampling irregular images. In [21], Li et al. showed a novel edge-orientation adaptive interpolation scheme. Their idea is to use the geometric duality of LR and HR covariance coefficients for image interpolation. In [22], Zhang et al. applied the fractal analysis method to the field of image magnification, and proposed a novel single-image super-resolution procedure based upon the rational fractal interpolation. In [23], Hussain et al. used a class of quadratic triangle B-spline with control parameters to implement image interpolation, and creatively applied genetic algorithms to optimize the selection of parameters, so as to obtain better quality interpolation images.

The purpose of this paper is to propose the improved first class of Side-Side (FCSS), second class of Side-Side (SCSS), first class of Side-Vertex (FCSV) and second class of Side-Vertex (SCSV) interpolation operators using a new class of rational quadratic/linear trigonometric Hermite functions. We use these methods to construct Coons surface patches and implement *C*^1^ surface stitching. Based on the property that the surface shape can be adjusted through parameters, we introduce a center of mass function value control method, by which we can construct surface patches with the center of mass passing through a fixed point. Finally, we apply these proposed interpolation methods to image interpolation for upscaling the images with factor 2,3 and 4, and make comparisons between our methods and some existing image interpolation methods.

The rest of this paper is organized as follows. The definition and properties of a new class of rational quadratic/linear trigonometric Hermite functions are described in Section 2. In Section 3, we discuss the four improved interpolation operators: FCSS, SCSS, FCSV and SCSV. The constructed Coons surface patches and stitched surfaces are showed in Section 4. Section 5 shows a center of mass function value control method. In Section 6, four interpolation methods are applied to image interpolation. Section 7 summarizes our work.

## 2.Trigonometric Hermite functions with shape parameters

For u ∈ [0, 1], we construct a new class of rational quadratic/linear trigonometric Hermite functions with two shape parameters *α*, *β* ∈ (0, + ∞) as follow

$$\begin{array}{c} \left\{ \begin{array}{c} T_{0}\left(u\right)=\frac{\alpha (1-\text{sin}\frac{\pi u}{2})}{\alpha \left(1-\text{sin}\frac{\pi u}{2}\right)+\beta \left(1-\text{cos}\frac{\pi u}{2}\right)},\\ T_{1}\left(u\right)=\frac{\beta (1-\text{cos}\frac{\pi u}{2})}{\alpha \left(1-\text{sin}\frac{\pi u}{2}\right)+\beta \left(1-\text{cos}\frac{\pi u}{2}\right)},\\ T_{2}\left(u\right)=\frac{2\alpha \text{sin}\frac{\pi u}{2}\left(1-\text{sin}\frac{\pi u}{2}\right)}{\pi \alpha \left(1-\text{sin}\frac{\pi u}{2}\right)+\pi \beta \left(1-\text{cos}\frac{\pi u}{2}\right)},\\ T_{3}\left(u\right)=-\frac{2\beta \text{cos}\frac{\pi u}{2}\left(1-\text{cos}\frac{\pi u}{2}\right)}{\pi \alpha \left(1-\text{sin}\frac{\pi u}{2}\right)+\pi \beta \left(1-\text{cos}\frac{\pi u}{2}\right)}. \end{array}\right. \end{array}$$ (1)

It is easy to check that *T*_0_(*u*)+*T*_1_(*u*) ≡ 1. And the trigonometric Hermite functions *T*_*i*_(*u*)(*i* = 0, 1, 2, 3) have the following important end-point properties

$$\begin{array}{c} \left\{ \begin{array}{c} T_{0}\left(0\right)=1,T_{0}\left(1\right)=0,T_{0}^{\prime }\left(0\right)=0,T_{0}^{\prime }\left(1\right)=0,\\ T_{1}\left(0\right)=0,T_{1}\left(1\right)=1,T_{1}^{\prime }\left(0\right)=0,T_{1}^{\prime }\left(1\right)=0,\\ T_{2}\left(0\right)=0,T_{2}\left(1\right)=0,T_{2}^{\prime }\left(0\right)=1,T_{2}^{\prime }\left(1\right)=0,\\ T_{3}\left(0\right)=0,T_{3}\left(1\right)=0,T_{3}^{\prime }\left(0\right)=0,T_{3}^{\prime }\left(1\right)=1. \end{array}\right. \end{array}$$

## 3.The improved Side-Side methods and Side-Vertex methods

Over triangular domain, we use transfinite interpolant to construct Coons surface patches. In order to facilitate the discussion over triangular domain, we first need to convert the Cartesian coordinates to the barycentric coordinates.

### 3.1 Barycentric coordinates

**Fig 1:**
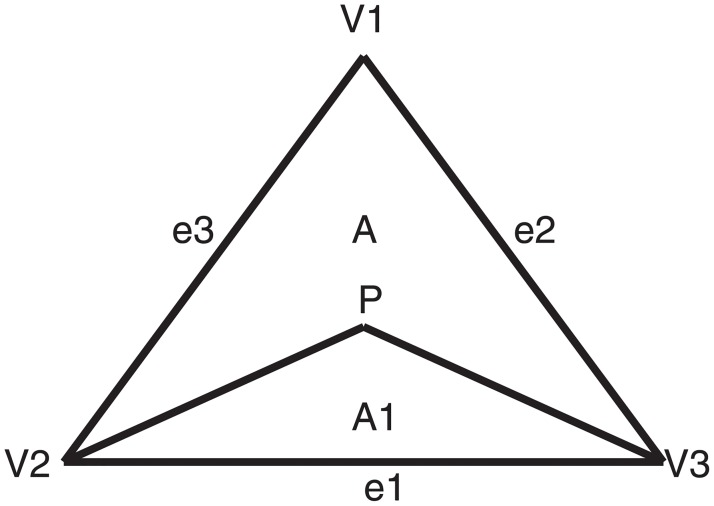
Triangle *T*.

Let the non-degenerate triangle be *T*, and its vertices be *V*_*i*_(*x*_*i*_, *y*_*i*_)(*i* = 1, 2, 3) counterclockwise (see Fig 1). ∂*T* represents the boundary of *T*. The vectors of three boundaries are marked as *e*_1_ = *V*_3_ − *V*_2_, *e*_2_ = *V*_1_ − *V*_3_, *e*_3_ = *V*_2_ − *V*_1_. For any point *P* inside *T*, the barycentric coordinates is (*b*_1_, *b*_2_, *b*_3_), where $b_{i}=\frac{A_{i}}{A}$, *A* is the area of *T*, and *A*_*i*_ is the area of Δ*PV*_*j*_ *V*_*k*_. *F* represents the continuous function on *T*.

The conversion relationship between the Cartesian coordinates (*x*, *y*) and the barycentric coordinates (*b*_1_, *b*_2_, *b*_3_) is as follows

$$\begin{array}{c} \left\{ \begin{array}{c} x=b_{1}x_{1}+b_{2}x_{2}+b_{3}x_{3},\\ y=b_{1}y_{1}+b_{2}y_{2}+b_{3}y_{3},\\ 1=b_{1}+b_{2}+b_{3}. \end{array}\right. \end{array}$$

### 3.2 The First Class of Side-Side (FCSS) interpolation operations

**Fig 2:**
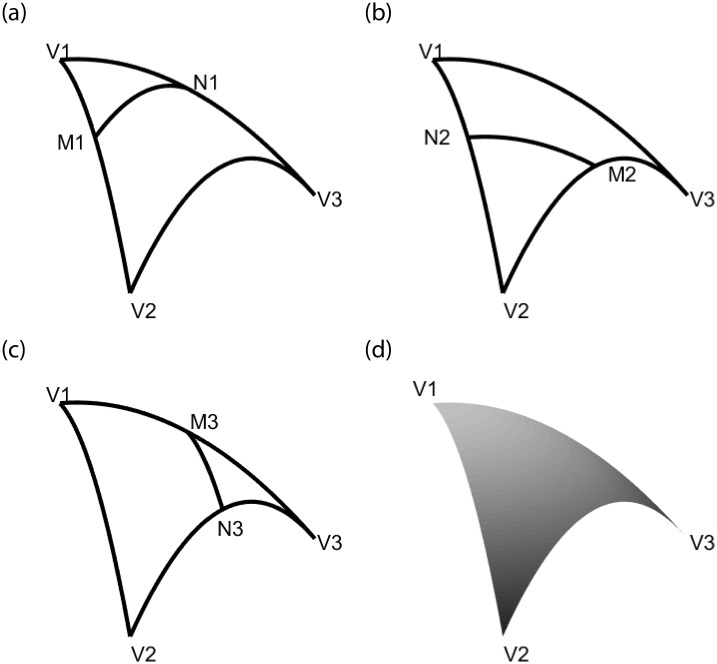
The geometric principle of Side-Side method. (a) The geometric principle of interpolation operator *P*_1_[*F*]. (b) The geometric principle of interpolation operator *P*_2_[*F*]. (c) The geometric principle of interpolation operator *P*_3_[*F*]. (d) The Coons surface patch obtained by Side-Side method.

The classic Side-Side method, proposed by Barnhill, Birkhoff and Gordon [4], is also called the parallel projection method. Each interpolation operator *P*_*i*_[*F*] represents the linear interpolation along segments parallel to the *ith* side of *T*. The geometric principle of the Side-Side method is shown in Fig 2.

Now we make an improvement to the first class of Side-Side interpolation operators. The Hermite functions in the operators are replaced by the new class of rational quadratic/linear trigonometric Hermite functions given in Eq (1), and the first class of Side-Side (FCSS for short) interpolation operators *P*_*i*_[*F*](*i* = 1, 2, 3) are defined as follows

$$\begin{array}{c} \begin{array}{cc} P_{1}\left[F\right] & =T_{0}\left(\frac{b_{3}}{1-b_{1}}\right)F\left(M_{1}\right)+T_{1}\left(\frac{b_{3}}{1-b_{1}}\right)F\left(N_{1}\right)\\ & +T_{2}\left(\frac{b_{3}}{1-b_{1}}\right)\left(1-b_{1}\right)\frac{\partial F}{\partial e_{1}}\left(M_{1}\right)+T_{3}\left(\frac{b_{3}}{1-b_{1}}\right)\left(1-b_{1}\right)\frac{\partial F}{\partial e_{1}}\left(N_{1}\right), \end{array} \end{array}$$ (2)

where

$$\begin{array}{c} M_{1}=b_{1}V_{1}+\left(1-b_{1}\right)V_{2},N_{1}=b_{1}V_{1}+\left(1-b_{1}\right)V_{3}, \end{array}$$

and $\frac{\partial F}{\partial e_{1}}\left(x\right)$ is the partial derivative of *F* along the direction of *e*_1_ at point *x*.

$$\begin{array}{c} \begin{array}{cc} P_{2}\left[F\right] & =T_{0}\left(\frac{b_{1}}{1-b_{2}}\right)F\left(M_{2}\right)+T_{1}\left(\frac{b_{1}}{1-b_{2}}\right)F\left(N_{2}\right)\\ & +T_{2}\left(\frac{b_{1}}{1-b_{2}}\right)\left(1-b_{2}\right)\frac{\partial F}{\partial e_{2}}\left(M_{2}\right)+T_{3}\left(\frac{b_{1}}{1-b_{2}}\right)\left(1-b_{2}\right)\frac{\partial F}{\partial e_{2}}\left(N_{2}\right), \end{array} \end{array}$$ (3)

where

$$\begin{array}{c} M_{2}=b_{2}V_{2}+\left(1-b_{2}\right)V_{3},N_{2}=b_{2}V_{2}+\left(1-b_{2}\right)V_{1}, \end{array}$$

and $\frac{\partial F}{\partial e_{2}}\left(x\right)$ is the partial derivative of *F* along the direction of *e*_2_ at point *x*.

$$\begin{array}{c} \begin{array}{cc} P_{3}\left[F\right] & =T_{0}\left(\frac{b_{2}}{1-b_{3}}\right)F\left(M_{3}\right)+T_{1}\left(\frac{b_{2}}{1-b_{3}}\right)F\left(N_{3}\right)\\ & +T_{2}\left(\frac{b_{2}}{1-b_{3}}\right)\left(1-b_{3}\right)\frac{\partial F}{\partial e_{3}}\left(M_{3}\right)+T_{3}\left(\frac{b_{2}}{1-b_{3}}\right)\left(1-b_{3}\right)\frac{\partial F}{\partial e_{3}}\left(N_{3}\right), \end{array} \end{array}$$ (4)

where

$$\begin{array}{c} M_{3}=b_{3}V_{3}+\left(1-b_{3}\right)V_{1},N_{3}=b_{3}V_{3}+\left(1-b_{3}\right)V_{2}, \end{array}$$

and $\frac{\partial F}{\partial e_{3}}\left(x\right)$ is the partial derivative of *F* along the direction of *e*_3_ at point *x*.

### 3.3 The second class of Side-Side (SCSS) interpolation operations

The second class of Side-Side interpolation operators also can be obtained by replacing the Hermite functions with two trigonometric Hermite functions *T*_1_(*u*) and *T*_3_(*u*) given in Eq (1), respectively. And the second class of Side-Side (SCSS for short) interpolation operators *P*_*i*_[*F*](*i* = 1, 2, 3) are defined as follows

$$\begin{array}{c} \begin{array}{cc} P_{1}\left[F\right] & =T_{1}\left(\frac{b_{3}}{1-b_{1}}\right)F\left(N_{1}\right)+T_{1}\left(1-\frac{b_{3}}{1-b_{1}}\right)F\left(M_{1}\right)\\ & \;+T_{3}\left(\frac{b_{3}}{1-b_{1}}\right)\left(1-b_{1}\right)\frac{\partial F}{\partial e_{1}}\left(N_{1}\right)-T_{3}\left(1-\frac{b_{3}}{1-b_{1}}\right)\left(1-b_{1}\right)\frac{\partial F}{\partial e_{1}}\left(M_{1}\right), \end{array} \end{array}$$ (5)

$$\begin{array}{c} \begin{array}{cc} P_{2}\left[F\right] & =T_{1}\left(\frac{b_{1}}{1-b_{2}}\right)F\left(N_{2}\right)+T_{1}\left(1-\frac{b_{1}}{1-b_{2}}\right)F\left(M_{2}\right)\\ & \;+T_{3}\left(\frac{b_{1}}{1-b_{2}}\right)\left(1-b_{2}\right)\frac{\partial F}{\partial e_{2}}\left(N_{2}\right)-T_{3}\left(1-\frac{b_{1}}{1-b_{2}}\right)\left(1-b_{2}\right)\frac{\partial F}{\partial e_{2}}\left(M_{2}\right), \end{array} \end{array}$$ (6)

$$\begin{array}{c} \begin{array}{cc} P_{3}\left[F\right] & =T_{1}\left(\frac{b_{2}}{1-b_{3}}\right)F\left(N_{3}\right)+T_{1}\left(1-\frac{b_{2}}{1-b_{3}}\right)F\left(M_{3}\right)\\ & \;+T_{3}\left(\frac{b_{2}}{1-b_{3}}\right)\left(1-b_{3}\right)\frac{\partial F}{\partial e_{3}}\left(N_{3}\right)-T_{3}\left(1-\frac{b_{2}}{1-b_{3}}\right)\left(1-b_{3}\right)\frac{\partial F}{\partial e_{3}}\left(M_{3}\right), \end{array} \end{array}$$ (7)

where the definitions of *M*_*i*_,*N*_*i*_,$\frac{\partial F}{\partial e_{i}}\left(i=1,2,3\right)$ are the same as (2), (3), and (4).

### 3.4 The First Class of Side-Vertex (FCSV) interpolation operations

**Fig 3:**
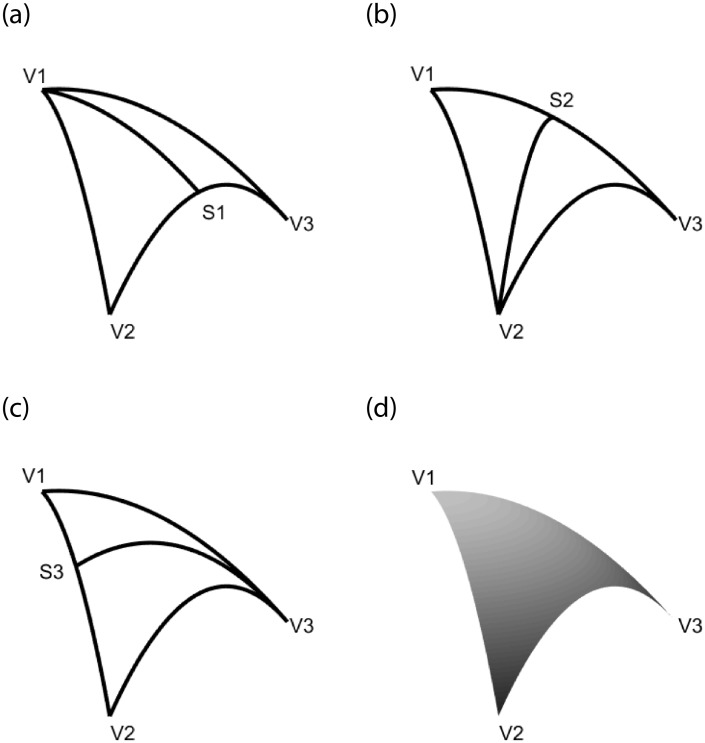
The geometric principle of Side-Vertex method. (a) The geometric principle of interpolation operator *Q*_1_[*F*]. (b) The geometric principle of interpolation operator *Q*_2_[*F*]. (c) The geometric principle of interpolation operator *Q*_3_[*F*]. (d) The Coons surface patch obtained by Side-Vertex method.

The Side-Vertex method proposed by Nielson [7] is also called the radial projection method. Each operator represents the interpolation along line joining a vertex and its opposing side. The principle of Side-Vertex method is shown in Fig 3.

Set *S*_*i*_ as the point on the boundary corresponding to vertex *V*_*i*_, and the coordinates of *S*_*i*_ is

$$\begin{array}{c} S_{i}=S_{i}\left(x,y\right)=\left(\frac{x-b_{i}x_{i}}{1-b_{i}},\frac{y-b_{i}y_{i}}{1-b_{i}}\right)=\left(\frac{b_{j}x_{j}+b_{k}x_{k}}{b_{j}+b_{k}},\frac{b_{j}y_{j}+b_{k}y_{k}}{b_{j}+b_{k}}\right). \end{array}$$ (8)

We also make an improvement to the first class of Side-Vertex interpolation operators. The Hermite functions are replaced by the trigonometric Hermite functions given in Eq (1), and the first class of Side-Vertex (FCSV for short) interpolation operators *Q*_*i*_[*F*](*i* = 1, 2, 3) are showed as follows

$$\begin{array}{c} Q_{1}\left[F\right]=T_{0}\left(b_{1}\right)F\left(S_{1}\right)+T_{2}\left(b_{1}\right)R_{1}^{\prime }\left(1\right)+T_{1}\left(b_{1}\right)F\left(V_{1}\right)+T_{3}\left(b_{1}\right)R_{1}^{\prime }\left(0\right), \end{array}$$ (9)

where $R_{1}^{\prime }\left(1\right)=\frac{b_{2}\frac{\partial F}{\partial e_{3}}\left(S_{1}\right)+b_{3}\frac{\partial F}{\partial e_{2}}\left(S_{1}\right)}{b_{2}+b_{3}},R_{1}^{\prime }\left(0\right)=\frac{b_{2}\frac{\partial F}{\partial e_{3}}\left(V_{1}\right)+b_{3}\frac{\partial F}{\partial e_{2}}\left(V_{1}\right)}{b_{2}+b_{3}}$.

$$\begin{array}{c} Q_{2}\left[F\right]=T_{0}\left(b_{2}\right)F\left(S_{2}\right)+T_{2}\left(b_{2}\right)R_{2}^{\prime }\left(1\right)+T_{1}\left(b_{2}\right)F\left(V_{2}\right)+T_{3}\left(b_{2}\right)R_{2}^{\prime }\left(0\right), \end{array}$$ (10)

where $R_{2}^{\prime }\left(1\right)=\frac{b_{3}\frac{\partial F}{\partial e_{1}}\left(S_{2}\right)+b_{1}\frac{\partial F}{\partial e_{3}}\left(S_{2}\right)}{b_{1}+b_{3}},R_{2}^{\prime }\left(0\right)=\frac{b_{3}\frac{\partial F}{\partial e_{1}}\left(V_{2}\right)+b_{1}\frac{\partial F}{\partial e_{3}}\left(V_{2}\right)}{b_{1}+b_{3}}$.

$$\begin{array}{c} Q_{3}\left[F\right]=T_{0}\left(b_{3}\right)F\left(S_{3}\right)+T_{2}\left(b_{3}\right)R_{3}^{\prime }\left(1\right)+T_{1}\left(b_{3}\right)F\left(V_{3}\right)+T_{3}\left(b_{3}\right)R_{3}^{\prime }\left(0\right), \end{array}$$ (11)

where $R_{3}^{\prime }\left(1\right)=\frac{b_{1}\frac{\partial F}{\partial e_{2}}\left(S_{3}\right)+b_{2}\frac{\partial F}{\partial e_{1}}\left(S_{3}\right)}{b_{1}+b_{2}},R_{3}^{\prime }\left(0\right)=\frac{b_{1}\frac{\partial F}{\partial e_{2}}\left(V_{3}\right)+b_{2}\frac{\partial F}{\partial e_{1}}\left(V_{3}\right)}{b_{1}+b_{2}}$.

### 3.5 The second class of Side-Vertex (SCSV) interpolation operations

By using the new trigonometric Hermite functions *T*_1_(*u*) and *T*_3_(*u*) given in Eq (1), the second class of Side-Vertex (SCSV for short) interpolation operators *Q*_*i*_[*F*](*i* = 1, 2, 3) are defined as follows

$$\begin{array}{c} Q_{1}\left[F\right]=T_{1}\left(1-b_{1}\right)F\left(S_{1}\right)+T_{3}\left(1-b_{1}\right)R_{1}^{\prime }\left(1\right)+T_{1}\left(b_{1}\right)F\left(V_{1}\right)-T_{3}\left(b_{1}\right)R_{1}^{\prime }\left(0\right), \end{array}$$ (12)

$$\begin{array}{c} Q_{2}\left[F\right]=T_{1}\left(1-b_{2}\right)F\left(S_{2}\right)+T_{3}\left(1-b_{2}\right)R_{2}^{\prime }\left(1\right)+T_{1}\left(b_{2}\right)F\left(V_{2}\right)-T_{3}\left(b_{2}\right)R_{2}^{\prime }\left(0\right), \end{array}$$ (13)

$$\begin{array}{c} Q_{3}\left[F\right]=T_{1}\left(1-b_{3}\right)F\left(S_{3}\right)+T_{3}\left(1-b_{3}\right)R_{3}^{\prime }\left(1\right)+T_{1}\left(b_{3}\right)F\left(V_{3}\right)-T_{3}\left(b_{3}\right)R_{3}^{\prime }\left(0\right). \end{array}$$ (14)

where the definitions of $R_{i}^{\prime }\left(1\right),R_{i}^{\prime }\left(0\right)\left(i=1,2,3\right)$ are the same as Eqs (9)–(11).

## 4.*C*^1^ Coons surfaces over triangular domain

### 4.1 Construction of Coons surfaces using Side-Side methods

Given the FCSS and SCSS interpolation operators *P*_*i*_[*F*](*i* = 1, 2, 3), the Coons surface patch is defined by the Boolean sum of three operators

$$\begin{array}{c} P\left[F\right]=\omega_{1}P_{1}\left[F\right]+\omega_{2}P_{2}\left[F\right]+\omega_{3}P_{3}\left[F\right], \end{array}$$ (15)

where

$$\begin{array}{c} \omega_{i}=\frac{b_{i}^{2}}{\sum_{m=1}^{3}b_{m}^{2}}. \end{array}$$ (16)

**Theorem 1**. *The weight function ω*_*i*_(*i* = 1, 2, 3) *given in* Eq (16) *has the following properties*:

1. *ω*_1_ + *ω*_2_ + *ω*_3_ = 1.
2. *ω*_*i*_|*e*_*i*_ = 0.
3. *For i* = 1, 2, 3, *j* = 1, 2, 3, $\frac{\partial \omega_{i}}{\partial \vec{e_{j}}}{|}_{e_{i}}=0$.

*Proof of Theorem 1*. Through simple calculations, we have

1. $\omega_{1}+\omega_{2}+\omega_{3}=\frac{b_{1}^{2}+b_{2}^{2}+b_{3}^{2}}{\sum_{m=1}^{3}b_{m}^{2}}=1$.
2. Obviously, *b*_*i*_ = 0 on the side *e*_*i*_, so
  $$\begin{array}{c} \omega_{i}{|}_{e_{i}}=\frac{b_{i}}{\sum_{m=1}^{3}b_{m}^{2}}{|}_{e_{i}}=0. \end{array}$$
3. Taking *i* = 1 as an example. Since in Barycentric coordinates, $\vec{e_{1}}=V_{3}-V_{2}=\left(0,-1,1\right)$, $\vec{e_{2}}=V_{1}-V_{3}=\left(1,0,-1\right)$, $\vec{e_{3}}=V_{2}-V_{1}=\left(-1,1,0\right)$ we have
  $$\begin{array}{c} \begin{array}{cc} \frac{\partial \omega_{1}}{\partial \vec{e_{1}}} & =\nabla \omega_{1}\cdot \vec{e_{1}}\\ & =(\frac{2b_{1}\left(b_{1}^{2}+b_{2}^{2}+b_{3}^{2}\right)-2b_{1}^{2}b_{1}}{{\left(b_{1}^{2}+b_{2}^{2}+b_{3}^{2}\right)}^{2}},-\frac{2b_{1}^{2}b_{2}}{{\left(b_{1}^{2}+b_{2}^{2}+b_{3}^{2}\right)}^{2}},-\frac{2b_{1}^{2}b_{3}}{{\left(b_{1}^{2}+b_{2}^{2}+b_{3}^{2}\right)}^{2}})\cdot (0,-1,1)\\ & =\frac{2b_{1}\left(b_{1}^{2}+b_{2}^{2}+b_{3}^{2}\right)-2b_{1}^{2}b_{1}}{{\left(b_{1}^{2}+b_{2}^{2}+b_{3}^{2}\right)}^{2}}\times 0+[-\frac{2b_{1}^{2}b_{2}}{{\left(b_{1}^{2}+b_{2}^{2}+b_{3}^{2}\right)}^{2}}]\times \left(-1\right)\\ & \;+[-\frac{2b_{1}^{2}b_{3}}{{\left(b_{1}^{2}+b_{2}^{2}+b_{3}^{2}\right)}^{2}}]\times 1\\ & =\frac{2b_{1}^{2}(b_{2}-b_{3})}{{\left(b_{1}^{2}+b_{2}^{2}+b_{3}^{2}\right)}^{2}}. \end{array} \end{array}$$
  $$\begin{array}{c} \begin{array}{cc} \frac{\partial \omega_{1}}{\partial \vec{e_{2}}} & =\nabla \omega_{1}\cdot \vec{e_{2}}\\ & =(\frac{2b_{1}\left(b_{1}^{2}+b_{2}^{2}+b_{3}^{2}\right)-2b_{1}^{2}b_{1}}{{\left(b_{1}^{2}+b_{2}^{2}+b_{3}^{2}\right)}^{2}},-\frac{2b_{1}^{2}b_{2}}{{\left(b_{1}^{2}+b_{2}^{2}+b_{3}^{2}\right)}^{2}},-\frac{2b_{1}^{2}b_{3}}{{\left(b_{1}^{2}+b_{2}^{2}+b_{3}^{2}\right)}^{2}})\cdot (1,0,-1)\\ & =\frac{2b_{1}\left(b_{1}^{2}+b_{2}^{2}+b_{3}^{2}\right)-2b_{1}^{2}b_{1}}{{\left(b_{1}^{2}+b_{2}^{2}+b_{3}^{2}\right)}^{2}}\times 1+[-\frac{2b_{1}^{2}b_{2}}{{\left(b_{1}^{2}+b_{2}^{2}+b_{3}^{2}\right)}^{2}}]\times 0\\ & \;+[-\frac{2b_{1}^{2}b_{3}}{{\left(b_{1}^{2}+b_{2}^{2}+b_{3}^{2}\right)}^{2}}]\times (-1)\\ & =\frac{2b_{1}b_{2}^{2}+2b_{1}b_{3}^{2}+2b_{3}b_{1}^{2}}{{\left(b_{1}^{2}+b_{2}^{2}+b_{3}^{2}\right)}^{2}}, \end{array} \end{array}$$
  $$\begin{array}{c} \begin{array}{cc} \frac{\partial \omega_{1}}{\partial \vec{e_{3}}} & =\nabla \omega_{1}\cdot \vec{e_{3}}\\ & =(\frac{2b_{1}\left(b_{1}^{2}+b_{2}^{2}+b_{3}^{2}\right)-2b_{1}^{2}b_{1}}{{\left(b_{1}^{2}+b_{2}^{2}+b_{3}^{2}\right)}^{2}},-\frac{2b_{1}^{2}b_{2}}{{\left(b_{1}^{2}+b_{2}^{2}+b_{3}^{2}\right)}^{2}},-\frac{2b_{1}^{2}b_{3}}{{\left(b_{1}^{2}+b_{2}^{2}+b_{3}^{2}\right)}^{2}})\cdot (-1,1,0)\\ & =\frac{2b_{1}\left(b_{1}^{2}+b_{2}^{2}+b_{3}^{2}\right)-2b_{1}^{2}b_{1}}{{\left(b_{1}^{2}+b_{2}^{2}+b_{3}^{2}\right)}^{2}}\times (-1)+[-\frac{2b_{1}^{2}b_{2}}{{\left(b_{1}^{2}+b_{2}^{2}+b_{3}^{2}\right)}^{2}}]\times 1\\ & \;+[-\frac{2b_{1}^{2}b_{3}}{{\left(b_{1}^{2}+b_{2}^{2}+b_{3}^{2}\right)}^{2}}]\times 0\\ & =\frac{-2b_{1}b_{2}^{2}-2b_{1}b_{3}^{2}-2b_{2}b_{1}^{2}}{{\left(b_{1}^{2}+b_{2}^{2}+b_{3}^{2}\right)}^{2}}. \end{array} \end{array}$$

From this together with *b*_1_ = 0 on the side *e*_1_, it follows immediately that $\frac{\partial \omega_{1}}{\partial \vec{e_{j}}}{|}_{e_{1}}=0$. The proofs for $\frac{\partial \omega_{2}}{\partial \vec{e_{j}}}{|}_{e_{2}}=0$ and $\frac{\partial \omega_{3}}{\partial \vec{e_{j}}}{|}_{e_{3}}=0$ are similar.

**Theorem 2**. *Let F*(*x*, *y*) ∈ *C*^1^(*T*), *when* (*x*, *y*) ∈ ∂*T*, *P*[*F*] *will interpolate F*(*x*, *y*) *and its first-order partial derivatives, so P*[*F*] ∈ *C*^1^(*T*).

*Proof of Theorem 2*. The following proof is based on the FCSS interpolation operators over triangular domain *T*. For *b*_*i*_ = 0 and *b*_*j*_ = 1 − *b*_*k*_ on the side *e*_*i*_, *i* = 1, 2, 3; *j* = 2, 3, 1; *k* = 3, 1, 2, the Boolean sum of three interpolation operators is

$$\begin{array}{c} P\left[F\right]{|}_{e_{i}}=\frac{b_{j}^{2}}{\sum_{m=0}^{3}b_{m}^{2}}P_{j}\left[F\right]+\frac{b_{k}^{2}}{\sum_{m=0}^{3}b_{m}^{2}}P_{k}\left[F\right], \end{array}$$

and

$$\begin{array}{c} \begin{array}{cc} P_{j}\left[F\right]{|}_{e_{i}} & =T_{0}\left(\frac{b_{i}}{1-b_{j}}\right)F\left(M_{j}\right)+T_{1}\left(\frac{b_{i}}{1-b_{j}}\right)F\left(N_{j}\right)\\ & +T_{2}\left(\frac{b_{i}}{1-b_{j}}\right)\left(1-b_{j}\right)\frac{\partial F}{\partial e_{j}}\left(M_{j}\right)+T_{3}\left(\frac{b_{i}}{1-b_{j}}\right)\left(1-b_{j}\right)\frac{\partial F}{\partial e_{j}}\left(N_{j}\right)\\ & =F(M_{j}), \end{array} \end{array}$$

$$\begin{array}{c} \begin{array}{cc} P_{k}\left[F\right]{|}_{e_{i}} & =T_{0}\left(\frac{b_{j}}{1-b_{k}}\right)F\left(M_{k}\right)+T_{1}\left(\frac{b_{j}}{1-b_{k}}\right)F\left(N_{k}\right)\\ & +T_{2}\left(\frac{b_{j}}{1-b_{k}}\right)\left(1-b_{k}\right)\frac{\partial F}{\partial e_{k}}\left(M_{k}\right)+T_{3}\left(\frac{b_{j}}{1-b_{k}}\right)\left(1-b_{k}\right)\frac{\partial F}{\partial e_{k}}\left(N_{k}\right)\\ & =F(N_{k}). \end{array} \end{array}$$

According to the geometric principle of Side-Side method shown in Fig 2, We find that *M*_*j*_ and *N*_*k*_ are points on the same boundary, thus

$$\begin{array}{c} P\left[F\right]{|}_{e_{i}}=\frac{b_{j}^{2}}{b_{j}^{2}+b_{k}^{2}}F\left(M_{j}\right)+\frac{b_{k}^{2}}{b_{j}^{2}+b_{k}^{2}}F\left(N_{k}\right)=F\left(S_{i}\right). \end{array}$$

Therefore, we have proved the following properties

$$\begin{array}{c} P\left[F\right]{|}_{\partial T}=F\left(x,y\right){|}_{\partial T}. \end{array}$$

Next, we prove that *P*[*F*] interpolates the first derivative values of the triangle boundaries. Taking *i* = 1 as an example, according to **Theorem** 1 (3), we have

$$\begin{array}{c} \begin{array}{cc} \frac{\partial P[F]}{\partial \vec{e_{1}}}{|}_{e_{1}} & =\frac{\partial (\omega_{1}P_{1}\left[F\right])}{\partial \vec{e_{1}}}{|}_{e_{1}}+\frac{\partial (\omega_{2}P_{2}\left[F\right])}{\partial \vec{e_{1}}}{|}_{e_{1}}+\frac{\partial (\omega_{3}P_{3}\left[F\right])}{\partial \vec{e_{1}}}{|}_{e_{1}}\\ & =\left(\frac{\partial \omega_{2}}{\partial \vec{e_{1}}}P_{2}\left[F\right]\right){|}_{e_{1}}+\left(\omega_{2}\frac{\partial P_{2}\left[F\right]}{\partial \vec{e_{1}}}\right){|}_{e_{1}}+\left(\frac{\partial \omega_{3}}{\partial \vec{e_{1}}}P_{3}\left[F\right]\right){|}_{e_{1}}+\left(\omega_{3}\frac{\partial P_{3}\left[F\right]}{\partial \vec{e_{1}}}\right){|}_{e_{1}}\\ & =\frac{-2b_{2}b_{3}^{2}-2b_{2}^{2}b_{3}}{{\left(b_{2}^{2}+b_{3}^{2}\right)}^{2}}F\left(M_{2}\right)+\frac{2b_{2}b_{3}^{2}+2b_{2}^{2}b_{3}}{{\left(b_{2}^{2}+b_{3}^{2}\right)}^{2}}F\left(N_{3}\right)\\ & \;+\frac{b_{2}^{2}}{{\left(b_{2}^{2}+b_{3}^{2}\right)}^{2}}\frac{\partial F(M_{2})}{\partial \vec{e_{1}}}+\frac{b_{3}^{2}}{{\left(b_{2}^{2}+b_{3}^{2}\right)}^{2}}\frac{\partial F(N_{3})}{\partial \vec{e_{1}}}\\ & =\frac{\partial F(S_{1})}{\partial \vec{e_{1}}}, \end{array} \end{array}$$

$$\begin{array}{c} \begin{array}{cc} \frac{\partial P[F]}{\partial \vec{e_{2}}}{|}_{e_{1}} & =\frac{\partial (\omega_{1}P_{1}\left[F\right])}{\partial \vec{e_{2}}}{|}_{e_{1}}+\frac{\partial (\omega_{2}P_{2}\left[F\right])}{\partial \vec{e_{2}}}{|}_{e_{1}}+\frac{\partial (\omega_{3}P_{3}\left[F\right])}{\partial \vec{e_{2}}}{|}_{e_{1}}\\ & =\left(\frac{\partial \omega_{2}}{\partial \vec{e_{2}}}P_{2}\left[F\right]\right){|}_{e_{1}}+\left(\omega_{2}\frac{\partial P_{2}\left[F\right]}{\partial \vec{e_{2}}}\right){|}_{e_{1}}+\left(\frac{\partial \omega_{3}}{\partial \vec{e_{2}}}P_{3}\left[F\right]\right){|}_{e_{1}}+\left(\omega_{3}\frac{\partial P_{3}\left[F\right]}{\partial \vec{e_{2}}}\right){|}_{e_{1}}\\ & =\frac{2b_{2}^{2}b_{3}}{{\left(b_{2}^{2}+b_{3}^{2}\right)}^{2}}F\left(M_{2}\right)+\frac{-2b_{2}^{2}b_{3}}{{\left(b_{2}^{2}+b_{3}^{2}\right)}^{2}}F\left(N_{3}\right)\\ & \;+\frac{b_{2}^{2}}{{\left(b_{2}^{2}+b_{3}^{2}\right)}^{2}}\frac{\partial F(M_{2})}{\partial \vec{e_{2}}}+\frac{b_{3}^{2}}{{\left(b_{2}^{2}+b_{3}^{2}\right)}^{2}}\frac{\partial F(N_{3})}{\partial \vec{e_{2}}}\\ & =\frac{\partial F(S_{1})}{\partial \vec{e_{2}}}, \end{array} \end{array}$$

$$\begin{array}{c} \begin{array}{cc} \frac{\partial P[F]}{\partial \vec{e_{3}}}{|}_{e_{1}} & =\frac{\partial (\omega_{1}P_{1}\left[F\right])}{\partial \vec{e_{3}}}{|}_{e_{1}}+\frac{\partial (\omega_{2}P_{2}\left[F\right])}{\partial \vec{e_{3}}}{|}_{e_{1}}+\frac{\partial (\omega_{3}P_{3}\left[F\right])}{\partial \vec{e_{3}}}{|}_{e_{1}}\\ & =\left(\frac{\partial \omega_{2}}{\partial \vec{e_{3}}}P_{2}\left[F\right]\right){|}_{e_{1}}+\left(\omega_{2}\frac{\partial P_{2}\left[F\right]}{\partial \vec{e_{3}}}\right){|}_{e_{1}}+\left(\frac{\partial \omega_{3}}{\partial \vec{e_{3}}}P_{3}\left[F\right]\right){|}_{e_{1}}+\left(\omega_{3}\frac{\partial P_{3}\left[F\right]}{\partial \vec{e_{3}}}\right){|}_{e_{1}}\\ & =\frac{2b_{2}b_{3}^{2}}{{\left(b_{2}^{2}+b_{3}^{2}\right)}^{2}}F\left(M_{2}\right)+\frac{-2b_{2}b_{3}^{2}}{{\left(b_{2}^{2}+b_{3}^{2}\right)}^{2}}F\left(N_{3}\right)\\ & \;+\frac{b_{2}^{2}}{{\left(b_{2}^{2}+b_{3}^{2}\right)}^{2}}\frac{\partial F(M_{2})}{\partial \vec{e_{3}}}+\frac{b_{3}^{2}}{{\left(b_{2}^{2}+b_{3}^{2}\right)}^{2}}\frac{\partial F(N_{3})}{\partial \vec{e_{3}}}\\ & =\frac{\partial F(S_{1})}{\partial \vec{e_{3}}}. \end{array} \end{array}$$

thus, we prove that

$$\begin{array}{c} \frac{\partial P[F]}{\partial \vec{e_{j}}}{|}_{e_{1}}=\frac{\partial F(S_{1})}{\partial \vec{e_{j}}}. \end{array}$$

Similarly, both on the side *e*_2_ and *e*_3_ of *T*, we can verify that

$$\begin{array}{c} \frac{\partial P[F]}{\partial \vec{e_{j}}}{|}_{e_{2}}=\frac{\partial F(S_{2})}{\partial \vec{e_{j}}},\frac{\partial P[F]}{\partial \vec{e_{j}}}{|}_{e_{3}}=\frac{\partial F(S_{3})}{\partial \vec{e_{j}}}. \end{array}$$

The proof of the SCSS interpolation operators is analogy to the FCSS interpolation operators. Therefore, we have verified that *P*[*F*] interpolates *F*(*x*, *y*) and its first-order partial derivatives of the triangle boundaries, which means that *P*[*F*] ∈ *C*^1^(*T*).

Both FCSS and SCSS interpolation operators contain two shape parameters *α* and *β*. Through adjusting the values of two shape parameters, we can obtain Coons surfaces of different shapes. Comparing to the classic Side-Side interpolation operators based on cubic Hermite basis functions, which construct the surface patches with fixed shape, both FCSS and SCSS interpolation operators show advantages in shape adjustment. Besides, **Theorem** 2 illustrates that no matter how the Coons surface is adjusted, function values and first-order partial derivatives of the triangle boundaries are always interpolated.

**Fig 4:**
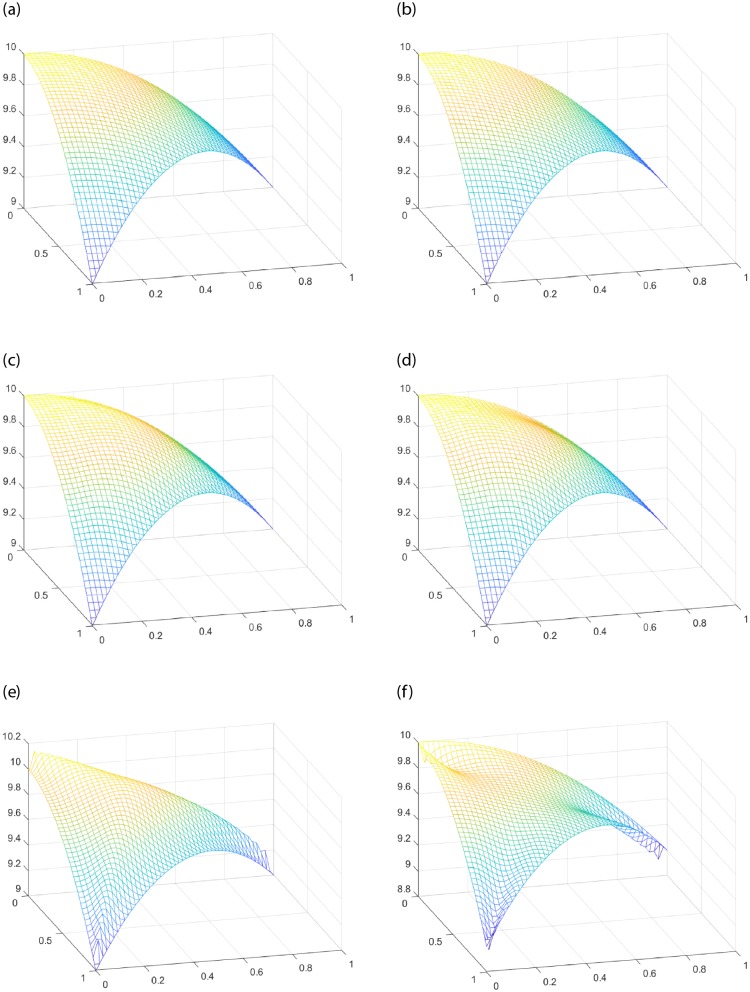
Comparison between the classic Side-Side interpolation operators and the improved parametric Side-Side methods to construct Coons surface patches. (a) The given function *f*(*x*, *y*). (b) The classic Side-Side interpolation operators. (c) FCSS with *α* = 1, *β* = 10. (d) FCSS with *α* = 10, *β* = 1. (e) SCSS with *α* = 1, *β* = 1.03. (f) SCSS with *α* = 1.03, *β* = 1.

Given the interpolating function *f*(*x*, *y*) = 10 − *x*^2^ − *y*^2^, the interpolating region *T* is a triangular domain with vertex *V*_1_(0, 0), *V*_2_(1, 0), *V*_3_(0, 1). For FCSS, when the shape parameters *α* > *β*, Fig 4d shows convex shape in the middle of the Coons surface patch. For SCSS, the effect of parameters on the shape is more obvious. When *α* < *β*, the surface patch in Fig 4e shows convex shape. When *α* > *β*, the surface patch Fig 4f presents concave shape. Fig 4 demonstrates that the interpolation surfaces can be adjusted conveniently by shape parameters. However, no matter how the shape changes, the Coons surface patches constructed by FCSS and SCSS still keep the interpolating property of **Theorem** 2.

Complex surface design can usually be achieved by splicing multiple small patches with simple shapes. Based on this technology, we first triangulate the area, use FCSS and SCSS interpolation operators to perform segmented transfinite interpolation, and finally stitch all the Coons patches to obtain a new surface.

**Fig 5:**
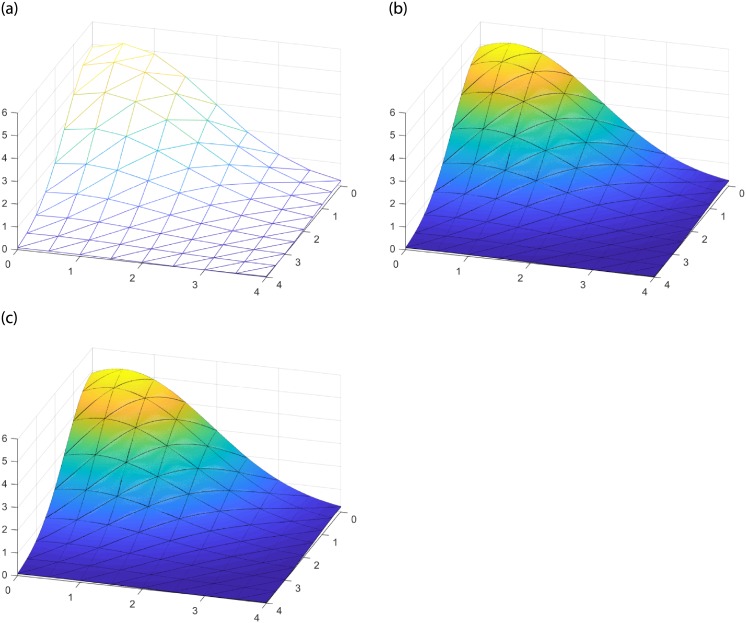
Splice surfaces constructed by FCSS and SCSS. (a) Triangulation. (b) FCSS with *α* = 1, *β* = 1. (c) SCSS with *α* = 1, *β* = 1.

Given the triangulation on Fig 5a and the surface function $g\left(x,y\right)=5.2\text{exp}\left[\frac{-x^{2}-{\left(y-0.5\right)}^{2}}{4}\right]$, (*x*, *y*) ∈ *R*^2^, Fig 5b and 5c display the splice surfaces constructed by the FCSS and SCSS interpolations operators respectively with shape parameters *α* = 1, *β* = 1. According to Theorem 2, the splice surfaces are *C*^1^ continuous. From Fig 5, we can see that the obtained stitching surfaces fit well.

### 4.2 Construction of Coons surfaces using Side-Vertex methods

Given the FCSV and SCSV interpolation operators *Q*_*i*_[*F*](*i* = 1, 2, 3; *j* = 2, 3, 1; *k* = 3, 1, 2), the construction of Coons surface patches is based upon the Boolean sum of three operators

$$\begin{array}{c} Q\left[F\right]=\bar{\omega_{1}}Q_{1}\left[F\right]+\bar{\omega_{2}}Q_{2}\left[F\right]+\bar{\omega_{3}}Q_{3}\left[F\right], \end{array}$$ (17)

where

$$\begin{array}{c} \bar{\omega_{i}}=\frac{b_{j}^{2}b_{k}^{2}}{b_{2}^{2}b_{3}^{2}+b_{1}^{2}b_{3}^{2}+b_{2}^{2}b_{1}^{2}}. \end{array}$$ (18)

**Theorem 3**. *The weight function* $\bar{\omega_{i}}\left(i=1,2,3\right)$ *given in* Eq (18) *has the following properties*:

1. $\bar{\omega_{1}}+\bar{\omega_{2}}+\bar{\omega_{3}}=1$.
2. $\bar{\omega_{i}}{|}_{e_{j}}=\delta_{ij}$.
3. For *i* = 1, 2, 3, *j* = 1, 2, 3, $\frac{\partial \bar{\omega_{i}}}{\partial \vec{e_{j}}}{|}_{e_{i}}=0$.

*Proof of Theorem 3*. The proof of **Theorem** 3 is analogy to **Theorem** 1.

**Theorem 4**. *Let F*(*x*, *y*) ∈ *C*^1^(*T*), *when* (*x*, *y*) ∈ ∂*T*, *Q*[*F*] *will interpolate* *F*(*x*, *y*) *and its first-order partial derivatives, so Q*[*F*] ∈ *C*^1^(*T*).

*Proof of Theorem 4*. The proof of **Theorem** 4 is analogy to **Theorem** 2.

The basis functions of two improved Side-Vertex methods also have shape parameters *α* and *β* analogous to the improved Side-Side methods, so we can adjust the shape by simply altering the parameter values, while the Coons surface patches still keep the interpolation property the same. Comparing to Nielson’s Side-Vertex method which is only suitable to construct fixed shape surfaces, the improved Side-Vertex methods have advantages.

**Fig 6:**
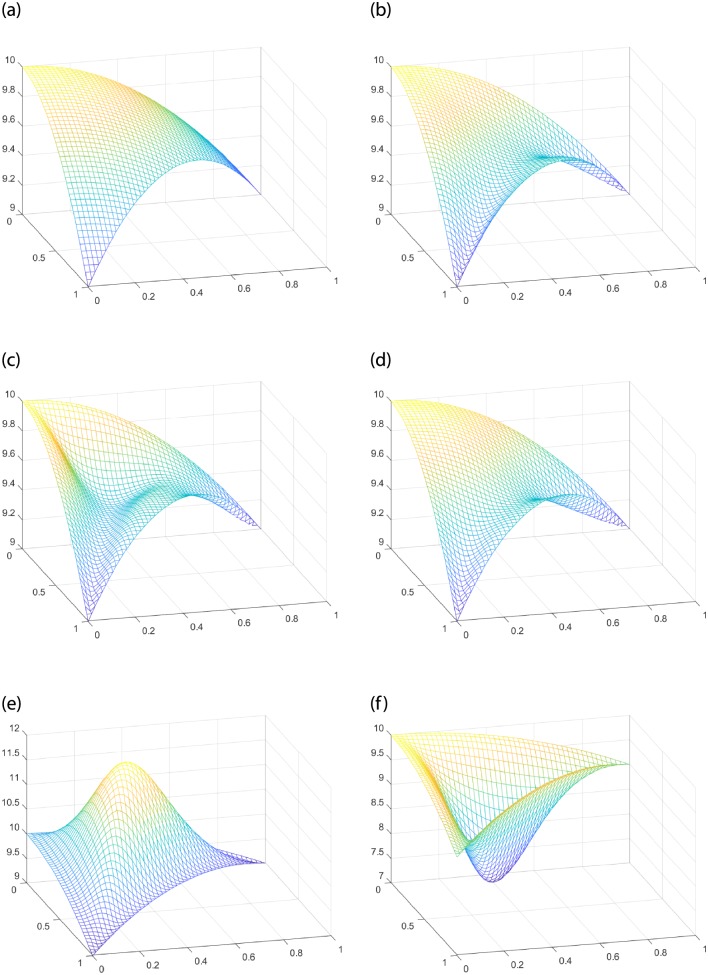
Comparison between Nielson’s Side-Vertex method and the improved parametric Side-Vertex methods to construct Coons surface patches. (a)The given function *f*(*x*, *y*). (b) Nielsion’s Side-Vertex method. (c) FCSV with *α* = 1, *β* = 10. (d) FCSV with *α* = 10, *β* = 1. (e) SCSV with *α* = 1, *β* = 2. (f) SCSV with *α* = 2, *β* = 1.

Given the function *f*(*x*, *y*) = 10 − *x*^2^ − *y*^2^, and the interpolating region *T* with *V*_1_(0, 0), *V*_2_(1, 0)*V*_3_(0, 1) the same as Fig 4, we can see that both FCSV and SCSV interpolations operators have good performances in changing the shape of the Coons surface patches from Fig 6. For FCSV, when the shape parameters *α* < *β*, the Coons surface patch presents concave shape (see Fig 6c). For SCSV, when *α* < *β*, the Coons surface patch presents convex shape (see Fig 6e). However, when *α* > *β*, it presents concave shape (see Fig 6f).

**Fig 7:**
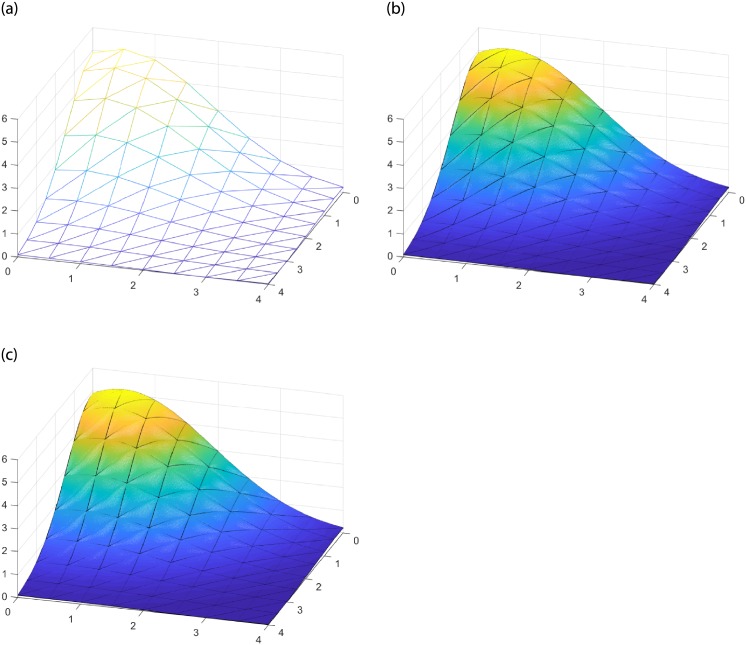
Splice surfaces constructed by FCSV and SCSV. (a) Triangulation. (b) FCSV with *α* = 1, *β* = 1. (c) SCSV with *α* = 1, *β* = 1.

Given the triangulation (see Fig 7a) and the interpolation function $g\left(x,y\right)=5.2\text{exp}\left[\frac{-x^{2}-{\left(y-0.5\right)}^{2}}{4}\right]$, (*x*, *y*) ∈ *R*^2^, Fig 7 shows the splice surfaces constructed by FCSV and SCSV with shape parameters *α* = 1, *β* = 1. When constructing surfaces with complex shape, we can apply this surface stitching method to achieve accurate reproduction.

### 4.3 Approximation precision of the improved Side-Side methods and Side-Vertex methods

**Fig 8:**
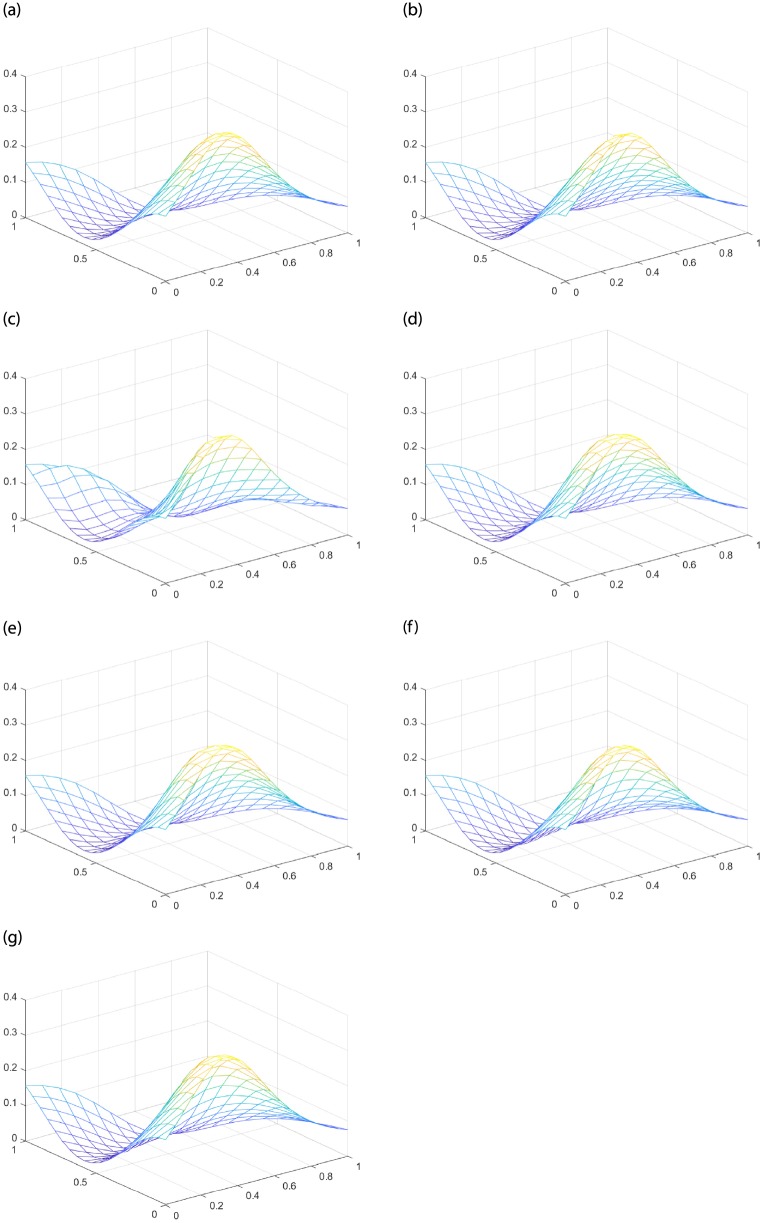
The original surface and reconstructed surfaces of *h*_3_. (a) Original surface. (b) The classic Side-Side method. (c) Nielson’s Side-Vertex method. (d) FCSS. (e) SCSS. (f) FCSV. (g) SCSV.

In order to demonstrate that the improved four interpolation methods have good approximation precision, we perform the following experiments. Given a test surface *F*(*x*, *y*), we first sample the boundary data, then apply the four improved interpolation methods to reconstruct interpolation surfaces and calculate the approximation accuracy of these restructured surfaces. We will also compare the approximation accuracy of the improved methods with the classic Side-Side method and Nielson’s Side-Vertex method. The four test surfaces in this experiment are constructed by the following functions

$$\begin{array}{c} \begin{array}{cc} h_{1}\left(x,y\right) & =0.75\;\text{exp}\left[-\frac{{\left(9x-2\right)}^{2}+{\left(9y-2\right)}^{2}}{4}\right]+0.75\;\text{exp}\left[-\frac{{\left(9x+1\right)}^{2}}{49}-\frac{9y+1}{10}\right]\\ & \;+0.5\;\text{exp}\left[-\frac{{\left(9x-7\right)}^{2}+{\left(9y-3\right)}^{2}}{4}\right]-0.2\;\text{exp}\left[-{\left(9x-4\right)}^{2}-{\left(9y-7\right)}^{2}\right],\\ h_{2}\left(x,y\right) & =\frac{\tanh(5-9x-9y)+1}{9},\\ h_{3}\left(x,y\right) & =\frac{1.25+\text{cos}(5.4y)}{6+6{\left(3x-1\right)}^{2}},\\ h_{4}\left(x,y\right) & =\frac{5.2\;\text{exp}(18y-18x)}{9\;\text{exp}(18y-18x)+9}, \end{array} \end{array}$$

where (*x*, *y*) ∈ *T* with *V*_1_(0, 0), *V*_2_(1, 0), *V*_3_(0, 1). The ranges of these functions are *h*_1_ ∈ [0, 1.220], *h*_2_ ∈ [0, 0.223], *h*_3_ ∈ [0, 0.375], *h*_4_ ∈ [0, 0.578]. Error between the reconstructed surface and the original surface is measured by root mean square (RMS)

$$\begin{array}{c} RMS=\sqrt{\frac{\sum_{i=0}^{M}\sum_{j=0}^{N}{\left(p\left(x_{i},y_{j}\right)-h\left(x_{i},y_{j}\right)\right)}^{2}}{\left(M+1)(N+1\right)}}, \end{array}$$

where *M* = *N* = 20, *p*(*x*_*i*_, *y*_*j*_) is the function value of interpolation surface, *h*(*x*_*i*_, *y*_*j*_) is the function value of original surface. To normalize the results, we divide RMS by the difference between the maximum and minimum values of *h* over *T*. In the following experiments, all *α* = 1, *β* = 1. Table 1 is the RMS errors of six interpolation methods: the classic Side-Side method, Nielson’s Side-Vertex method, FCSS, SCSS, FCSV and SCSV. Fig 8 is the original surface and restructured surfaces of *h*_3_.

**Table 1:** RMS errors of six interpolation methods.

	*h*_1_	*h*_2_	*h*_3_	*h*_4_
The classic Side-Side	0.078	0.049	0.021	0.066
Nielson’s Side-Vertex	0.126	0.080	0.071	0.066
FCSS	0.054	0.047	0.011	0.052
SCSS	0.054	0.047	0.011	0.052
FCSV	0.048	0.023	0.015	0.056
SCSV	0.048	0.023	0.011	0.056

Comparing the results of different interpolation methods in Table 1, regardless any shape of the test surface, the RMS errors of the improved Side-Side methods and Side-Vertex methods are less than the classic Side-Side method and Nielson’s Side-Vertex method, and less than 6 percent of the range of the function values. These results demonstrate that the four improved interpolation methods have higher approximation accuracy, and are more competitive than the classic Side-Side method and Nielson’s Side-Vertex method.

## 5.Center of mass function value control method

In practical applications, the center of mass over triangular domain is more important than other points for shape control. Since we can use shape parameters *α*, *β* to adjust the surface, we expect the control of the center of mass can also be achieved through parameters. The method introduced below can solve this issue perfectly.

If we require that the function value of the interpolation surface at the center of mass is equal to *M*, where $M\in \{\min_{1\leq i\leq 3}F\left(V_{i}\right),\max_{1\leq i\leq 3}F\left(V_{i}\right)\}$, we only need the shape parameters to satisfy:

$$\begin{array}{c} \begin{array}{cc} & P\left[F\right]=\omega_{1}P_{1}\left[F\right]+\omega_{2}P_{2}\left[F\right]+\omega_{3}P\left[F\right]=M,\\ or\; & Q\left[F\right]=\varpi_{1}Q_{1}\left[F\right]+\varpi_{2}Q_{2}\left[F\right]+\varpi_{3}Q_{3}\left[F\right]=M. \end{array} \end{array}$$ (19)

For convenience, we use a new variable *k* to represent the shape parameters *α*, *β*, where $k=\frac{\alpha }{\beta }$. The center of mass coordinates of the triangle is known to be $\left(\frac{1}{3},\frac{1}{3},\frac{1}{3}\right)$. By substituting the shape parameter ratio *k* and the coordinates into (19) and making a simplification, we can derive the following equations about *k*.

For FCSS, the equation of *k* is

$$\begin{array}{c} \{\pi \left[F\left(M_{1}\right)+F\left(M_{2}\right)+F\left(M_{3}\right)\right]+\frac{2\sqrt{2}}{3}\left[\frac{\partial F}{\partial e_{1}}\left(M_{1}\right)+\frac{\partial F}{\partial e_{2}}\left(M_{2}\right)+\frac{\partial F}{\partial e_{3}}\left(M_{3}\right)\right]-3\pi M\}k\\ =-\pi \left[F\left(N_{1}\right)+F\left(N_{2}\right)+F\left(N_{3}\right)\right]+\frac{2\sqrt{2}}{3}\left[\frac{\partial F}{\partial e_{1}}\left(N_{1}\right)+\frac{\partial F}{\partial e_{2}}\left(N_{2}\right)+\frac{\partial F}{\partial e_{3}}\left(N_{3}\right)\right]+3\pi M. \end{array}$$ (20)

For SCSS, the equation of *k* is

$$\begin{array}{c} \begin{array}{cc} & 3\pi Mk=\pi [F\left(N_{1}\right)+F\left(N_{2}\right)+F\left(N_{3}\right)+F\left(M_{1}\right)+F\left(M_{2}\right)+F\left(M_{3}\right)]\\ & \;+\frac{2\sqrt{2}}{3}\left[-\frac{\partial F}{\partial e_{1}}\left(N_{1}\right)-\frac{\partial F}{\partial e_{2}}\left(N_{2}\right)-\frac{\partial F}{\partial e_{3}}\left(N_{3}\right)+\frac{\partial F}{\partial e_{1}}\left(M_{1}\right)+\frac{\partial F}{\partial e_{2}}\left(M_{2}\right)+\frac{\partial F}{\partial e_{3}}\left(M_{3}\right)\right]-3\pi M. \end{array} \end{array}$$ (21)

For FCSV, the equation of *k* is

$$\begin{array}{c} \begin{array}{cc} & \{\pi \left[F\left(S_{1}\right)+F\left(S_{2}\right)+F\left(S_{3}\right)\right]+R_{1}\left(1\right)+R_{2}\left(1\right)+R_{3}\left(1\right)-3\pi M\}k\\ = & \;\left(\sqrt{3}-2\right)\pi \left[F\left(V_{1}\right)+F\left(V_{2}\right)+F\left(V_{3}\right)\right]\\ + & \;\sqrt{3}\left(2-\sqrt{3}\right)\left[R_{1}\left(0\right)+R_{2}\left(0\right)+R_{3}\left(0\right)\right]+3\pi \left(2-\sqrt{3}\right)M. \end{array} \end{array}$$ (22)

For SCSV, the equation of *k* is

$$\begin{array}{c} \begin{array}{cc} & \pi k\left[F\left(S_{1}\right)+F\left(S_{2}\right)+F\left(S_{3}\right)\right]-k\left[R_{1}\left(1\right)+R_{2}\left(1\right)+R_{3}\left(1\right)\right]\\ + & \left(7-4\sqrt{3}\right)\pi k\left[F\left(V_{1}\right)+F\left(V_{2}\right)+F\left(V_{3}\right)\right]+\left(7\sqrt{3}-12\right)k\left[R_{1}\left(0\right)+R_{2}\left(0\right)+R_{3}\left(0\right)\right]\\ - & 3[\left(2-\sqrt{3}\right)k^{2}+\left(8-4\sqrt{3}\right)k]\pi M\\ = & -\pi \left(2-\sqrt{3}\right)\left[F\left(S_{1}\right)+F\left(S_{2}\right)+F\left(S_{3}\right)\right]+\left(2-\sqrt{3}\right)\left[R_{1}\left(1\right)+R_{2}\left(1\right)+R_{3}\left(1\right)\right]\\ - & \left(2-\sqrt{3}\right)\pi \left[F\left(V_{1}\right)+F\left(V_{2}\right)+F\left(V_{3}\right)\right]-\left(2\sqrt{3}-3\right)\left[R_{1}\left(0\right)+R_{2}\left(0\right)+R_{3}\left(0\right)\right]+3\left(2-\sqrt{3}\right)\pi M. \end{array} \end{array}$$ (23)

By solving the above simple first or quadratic equation, the shape parameter ratio *k* of FCSS, SCSS, FCSV or SCSV can be obtained.

**Fig 9:**
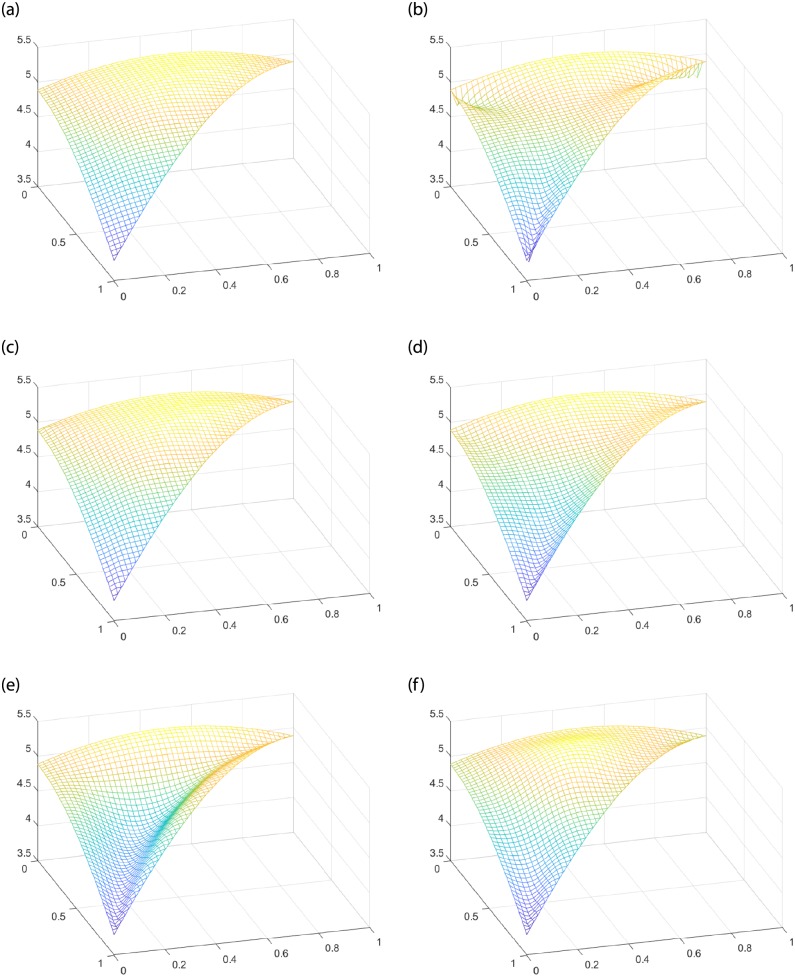
Coons surface patches with fixed function values on the center of mass. (a) FCSS. (b) SCSS_1. (c) SCSS_2. (d) FCSV. (e) FCSV_1. (f) FCSV_2.

Set three vertices of the triangle as *V*_1_(0, 0), *V*_2_(1, 0), *V*_3_(0, 1), and the value of center of mass as *M*. The boundaries of the surface patch are constructed by function $g\left(x,y\right)=5.2\text{exp}\left[\frac{-x^{2}-{\left(y-0.5\right)}^{2}}{4}\right]$, (*x*, *y*) ∈ *R*^2^. We use these four methods to construct Coons surface patches respectively. The given values *M* and the calculated shape parameter ratios are shown in Table 2. Fig 9 shows the improved Side-Side methods and Side-Vertex methods Coons surface patches with the corresponding *M* displayed in Table 2.

**Table 2:** Center of Mass function values and the parameter ratios *k*.

	FCSS	SCSS_1	SCSS_2	FCSV	SCSV_1	SCSV_2
*M*	5.11	4.87	5.14	4.80	4.39	5.22
*k*	1.01	1.10	0.99	1.05	1.32	0.78

## 6.Apply four new interpolation operators to image interpolation

In this section, we apply four new constructed FCSS, SCSS, FCSV and SCSV interpolation operators to image interpolation and achieve image magnification. The proposed methods are tested with six different standard images, which are gray scale and named as ‘Airplane’, ‘Baboon’, ‘Flower’, ‘Peppers’, ‘Rail’ (kodim19) and ‘Truck’ respectively. They are obtained from SIPI image database, University of Southern California [24] and the classic Kodak test image data set (http://r0k.us/graphics/kodak/). Besides, these methods will be compared with some classic schemes: bilinear interpolation, bicubic interpolation, kernel regression (KR) [20], new edge-directed interpolation (NEDI) [21], cubic trigonometric B-spline image interpolation scheme (CTB) [25] and rational cubic B-spline image interpolation scheme (RBC) [26]. The following algorithm illustrates the steps to implement image interpolation using the methods of this paper.

*Algorithm 1*.

*Input*. Standard test image, parameters *α* and *β*.

*Output*. Upscaling image, PSNR value and SSIM value.

*Step 1*. **Convert the input image to a gray matrix *G***. The elements *g*_*ij*_(*i* = 1, 2, …*m*, *j* = 1, 2, …, *n*) of matrix *G* represent the pixel values of corresponding pixel points in the *i* row and *j* column of the image.

*Step 2*. **Down-sample the original image to obtain low-resolution image**. Depending on whether the required interpolation factor is 2, 3 or 3, the pixel matrix of low resolution image is obtained by down-sampling the original image with interpolation factor 1/2 (sample one pixel every two pixels), 1/3 (sample one pixel every three pixels) or 1/4 (sample one pixel every four pixels). The grayscale matrix corresponding to the low resolution image is recorded as *G*_*d*_.

*Step 3*. **Use FCSS, SCSS, FCSV and SCSV for fragment interpolation**. Subdivide the pixel matrix *G*_*d*_ into small pixel matrices. Each small pixel matrix is 2 × 2, and stores the pixel values of a rectangular region in the down-sampled image. We divide the rectangular region into two triangle regions by triangulation and perform transfinite interpolation on each triangle patch. In order to implement the interpolation scheme, an approximation is made that the boundary values are considered as piecewise linear. After transfinite interpolation is done on all small matrices, we get the high resolution image. The gray matrix calculated by the interpolation method is recorded as $\bar{G}={\bar{g}}_{ij}\left(i=1,2,\ldots m,j=1,2,\ldots ,n\right)$.

*Step 4*. **Results analysis**. Compare the results of our four interpolation methods against some existing methods by analyzing the visual quality and calculating the values of Peak Signal to Noise Ratio (PSNR) and Structural Similarity Index (SSIM). PSNR is defined as

$$\begin{array}{c} PSNR=10{\text{log}}_{10}\left(\frac{255^{2}}{MSE}\right). \end{array}$$

where MSE is the mean square error defined as $MSE=\frac{1}{mn}\sum_{i=0}^{m}\sum_{j=0}^{n}|g_{ij}-{\bar{g}}_{ij}{|}^{2}$.

SSIM is defined as

$$\begin{array}{c} SSIM=\frac{\left(2\mu_{1}\mu_{2}+c_{1}\right)\left(2\sigma_{12}+c_{2}\right)}{\left({\mu_{1}}^{2}+{\mu_{2}}^{2}+c_{1}\right)\left({\sigma_{1}}^{2}+{\sigma_{2}}^{2}+c_{2}\right)}. \end{array}$$

where *μ*_1_ is the mean of *g*_*ij*_. *μ*_2_ is the mean of ${\bar{g}}_{ij}$. ${\sigma_{1}}^{2}$ is the variance of *g*_*ij*_. ${\sigma_{2}}^{2}$ is the variance of ${\bar{g}}_{ij}$. *σ*_12_ is the covariance of *g*_*ij*_ and ${\bar{g}}_{ij}$. *c*_1_ = (*k*_1_*L*)^2^, *c*_2_ = (*k*_2_*L*)^2^ are the constant used to maintain stability. *L* is the dynamic range of the pixel values. Generally *k*_1_ = 0.01, *k*_2_ = 0.03. Higher PSNR and SSIM mean better image quality.

When the image interpolation is performed by FCSS, SCSS, FCSV and SCSV, different interpolation results can be obtained by altering the values of parameters *α* and *β*, and interpolated images will have different qualities. However, based on **Theorems** 2 and 4, for the boundary values do not change with the shape parameters, the adjustment of the shape by parameters is limited to a reasonable range, which means that the adjusted image always maintains a high degree of fit with the original image, and a fine adjustment will result in a high quality image. We perform experiments on image enlargement with interpolation factors 2, 3 and 4, respectively. In the experiment of image magnification with interpolation factor 2, we compare our four methods with bilinear interpolation, bicubic interpolation, KR, NEDI, and CTB. Table 3 lists the values of parameters *α*, *β* used in all experiments. Tables 4 and 5 list the PSNR and SSIM values for each image interpolation scheme with interpolation factor 2. PSNR and SSIM data for KR and NEDI are referenced from [27].

**Table 3:** Parameter values.

	FCSS	SCSS	FCSV	SCSV
*α*	1	1.001	1	1
*β*	1	1	5	1

**Table 4:** PSNR values of different interpolation schemes (Interpolation Factor 2).

	bilinear	bicubic	KR	NEDI	CTB	FCSS	SCSS	FCSV	SCSV
Baboon	17.74	19.18	22.17	22.55	22.49	22.73	22.73	22.73	22.73
Peppers	21.26	24.93	29.22	29.72	30.54	31.31	31.31	31.31	31.31
Rail	17.98	20.80	21.79	22.78	24.59	25.52	25.52	25.52	25.52
Truck	24.40	27.41	31.36	32.13	31.43	32.19	32.19	32.19	32.19
Average	20.35	23.08	26.14	26.80	27.26	27.94	27.94	27.94	27.94

**Table 5:** SSIM values of different interpolation schemes (Interpolation Factor 2).

	bilinear	bicubic	KR	NEDI	CTB	FCSS	SCSS	FCSV	SCSV
Baboon	0.205	0.451	0.842	0.870	0.704	0.706	0.706	0.706	0.706
Peppers	0.620	0.753	0.954	0.965	0.855	0.866	0.866	0.866	0.866
Rail	0.493	0.693	0.722	0.793	0.835	0.845	0.845	0.845	0.845
Truck	0.466	0.684	0.932	0.946	0.847	0.854	0.853	0.854	0.854
Average	0.446	0.645	0.863	0.894	0.810	0.818	0.818	0.818	0.818

In terms of the data shown in Tables 4 and 5, the PSNR values of our four methods are the highest among all image interpolation schemes. In general, the PSNR values of our interpolation methods have pleasing results, that is, the error between corresponding pixels is small. The SSIM values of our methods are better than bilinear interpolation, bicubic interpolation and CTB. Although our methods are not as good as KR and NEDI in terms of SSIM, we can see the average SSIM values of our methods differ from KR by less than 0.05, and differ from NEDI by less than 0.08. Therefore, our methods still give pleasing results overall.

**Fig 10:**
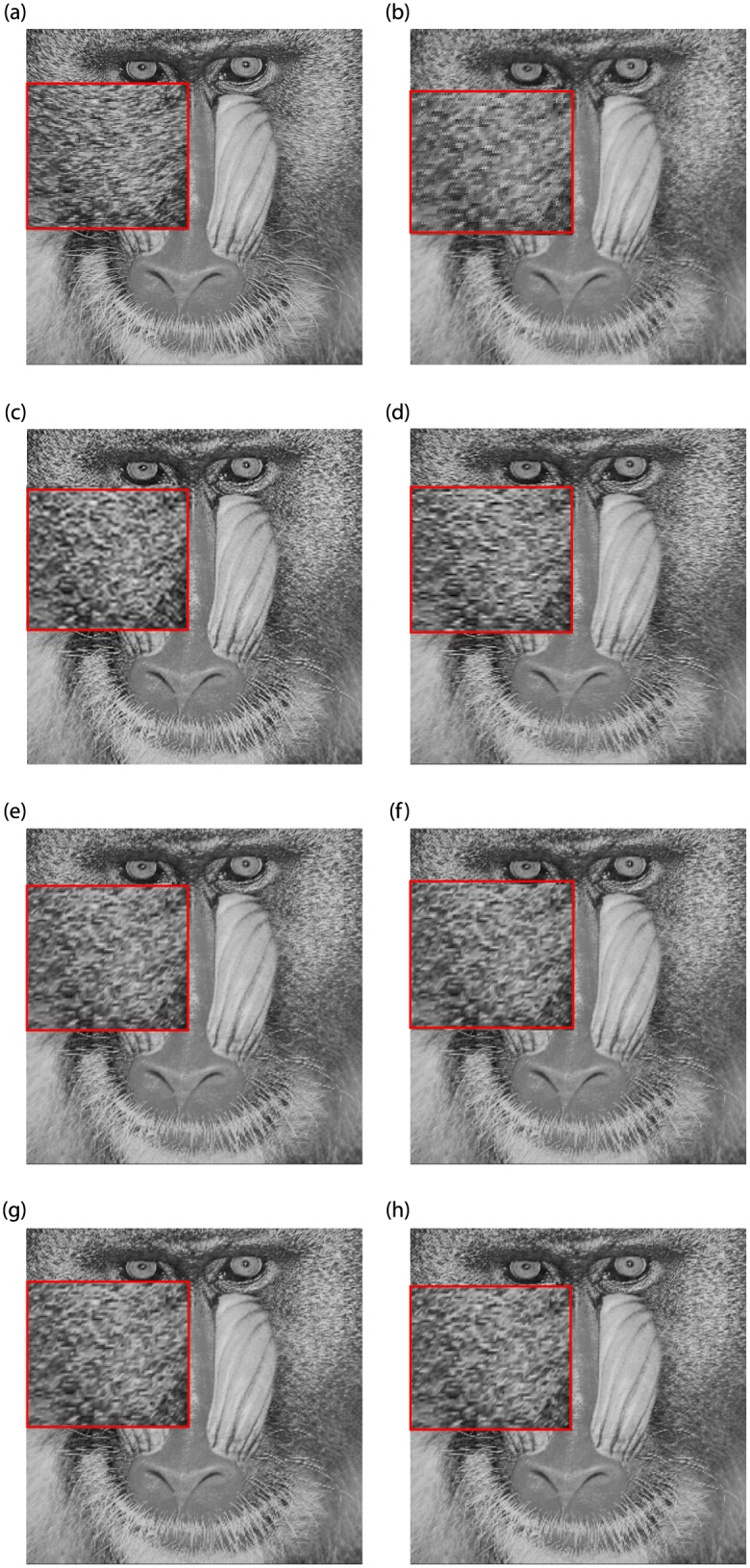
Comparison of Baboon images (Interpolation Factor 2). (a) Original image. (b) Bilinear interpolation. (c) Bicubic interpolation. (d) CTB. (e) FCSS. (f) SCSS. (g) FCSV. (h) SCSV.

**Fig 11:**
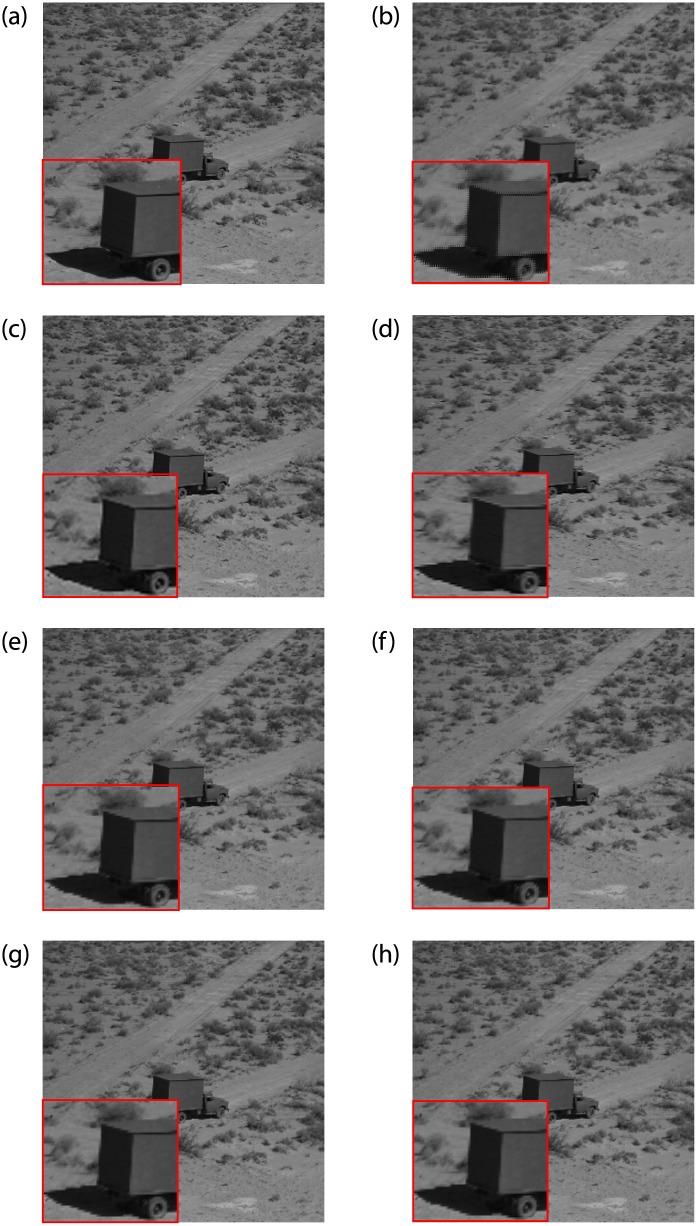
Comparison of Truck images (Interpolation Factor 2). (a) Original image. (b) Bilinear interpolation. (c) Bicubic interpolation. (d) CTB. (e) FCSS. (f) SCSS. (g) FCSV. (h) SCSV.

Figs 10 and 11 are upscaling images with interpolation factor 2 of seven methods: bilinear interpolation, bicubic interpolation, CTB, FCSS, SCSS, FCSV and SCSV. Comparing the visual quality of different interpolation schemes, for Fig 10b, bilinear interpolation shows the problem of damaging the texture of the image. In addition, bilinear interpolation is poor at edge preservation, where jaggy artifacts are observed alone the shadow edges, see Fig 11b. The image obtained by bicubic interpolation is obviously better than bilinear interpolation, and bicubic interpolation works well in maintaining edges. But comparing the texture direction in Fig 11a and 11c, we find that bicubic interpolation fails in preserving the texture direction. In terms of CTB, it has made a breakthrough in keeping the edges of the image. But looking at Fig 10d, we find that the reconstruction of the texture regions is still not perfect. The four methods we proposed overcome the problem of jagged edges to a certain extent. It can also be seen from Fig 10e–10h that these four improved interpolation methods preserve the texture direction and reproduce the texture details.

Furthermore, we also use FCSS, SCSS, FCSV and SCSV to upscale images with interpolation factor 3 and 4, and compare with NEDI and RBC. The standard test images are ‘Peppers’, ‘Airplane’ and ‘Flower’. In order to fully evaluate the quality of high resolution images, in addition to PSNR and SSIM, we have also added two image quality assessment metrics, which are MS-SSIM [28] and FSIM [29]. Similarly, the higher values of MS-SSIM and FSIM indicate the better quality of upscaling images. The PSNR, SSIM, MS-SSIM, FSIM results of NEDI and RBC are referenced from [26].

Tables 6–9 show PSNR, SSIM, MS-SSIM, and FSIM values of different image interpolation methods. For Tables 6–8, it can be noted that the PSNR, SSIM, and MS-SSIM of our four image interpolation methods are the highest. In Table 9, the FSIM of our method is higher than NEDI and slightly lower than RBC.

**Table 6:** PSNR values of different interpolation schemes (Interpolation Factor 3 and 4).

	Interpolation Factor 3						Interpolation Factor 4					
Images	NEDI	RBC	FCSS	SCSS	FCSV	SCSV	NEDI	RBC	FCSS	SCSS	FCSV	SCSV
Peppers	24.58	25.16	28.51	28.51	28.47	28.50	22.69	22.98	26.68	26.68	26.64	26.65
Airplane	26.58	27.43	27.74	27.74	27.63	27.68	24.88	25.79	25.33	25.33	25.21	25.28
Flower	30.76	31.48	32.56	32.56	32.42	32.47	28.59	29.64	29.64	29.64	29.44	29.54
Average	27.31	28.02	29.60	29.60	29.51	29.55	25.39	26.14	27.22	27.22	27.10	27.16

**Table 7:** SSIM values of different interpolation schemes (Interpolation Factor 3 and 4).

	Interpolation Factor 3						Interpolation Factor 4					
Images	NEDI	RBC	FCSS	SCSS	FCSV	SCSV	NEDI	RBC	FCSS	SCSS	FCSV	SCSV
Peppers	0.726	0.770	0.820	0.820	0.818	0.818	0.669	0.699	0.783	0.783	0.780	0.780
Airplane	0.845	0.862	0.876	0.876	0.874	0.874	0.806	0.823	0.818	0.818	0.812	0.815
Flower	0.838	0.854	0.924	0.924	0.923	0.923	0.789	0.812	0.881	0.881	0.876	0.879
Average	0.803	0.829	0.873	0.873	0.872	0.872	0.755	0.778	0.827	0.827	0.823	0.825

**Table 8:** MS-SSIM values of different interpolation schemes (Interpolation Factor 3 and 4).

	Interpolation Factor 3						Interpolation Factor 4					
Images	NEDI	RBC	FCSS	SCSS	FCSV	SCSV	NEDI	RBC	FCSS	SCSS	FCSV	SCSV
Peppers	0.929	0.931	0.965	0.965	0.964	0.964	0.869	0.881	0.948	0.947	0.946	0.946
Airplane	0.946	0.959	0.971	0.971	0.970	0.971	0.718	0.940	0.940	0.940	0.938	0.939
Flower	0.947	0.954	0.983	0.983	0.982	0.982	0.915	0.926	0.960	0.960	0.959	0.959
Average	0.941	0.948	0.973	0.973	0.972	0.972	0.834	0.916	0.949	0.949	0.948	0.948

**Table 9:** FSIM values of different interpolation schemes (Interpolation Factor 3 and 4).

	Interpolation Factor 3						Interpolation Factor 4					
Images	NEDI	RBC	FCSS	SCSS	FCSV	SCSV	NEDI	RBC	FCSS	SCSS	FCSV	SCSV
Peppers	0.965	0.983	0.957	0.957	0.956	0.956	0.958	0.970	0.933	0.933	0.932	0.933
Airplane	0.941	0.971	0.952	0.952	0.951	0.951	0.943	0.952	0.910	0.910	0.907	0.909
Flower	0.936	0.961	0.975	0.975	0.974	0.975	0.936	0.954	0.952	0.952	0.950	0.951
Average	0.947	0.972	0.961	0.961	0.960	0.961	0.946	0.959	0.932	0.932	0.930	0.931

**Fig 12:**
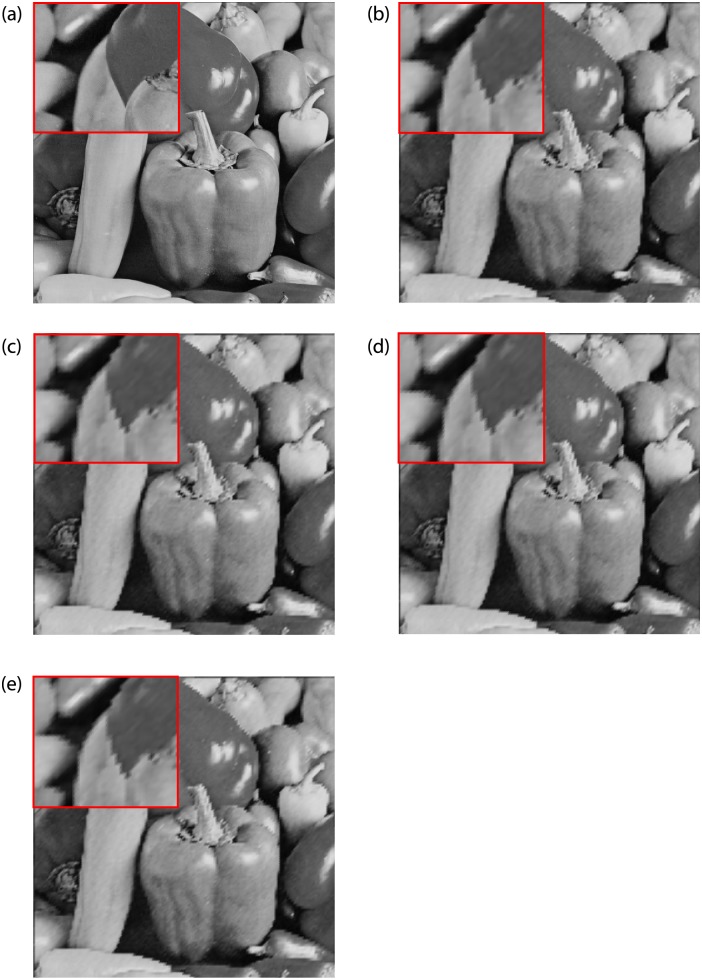
Comparison of Peppers images (Interpolation Factor 4). (a) Original image. (b) FCSS. (c) SCSS. (d) FSCV (e) SCSV.

**Fig 13:**
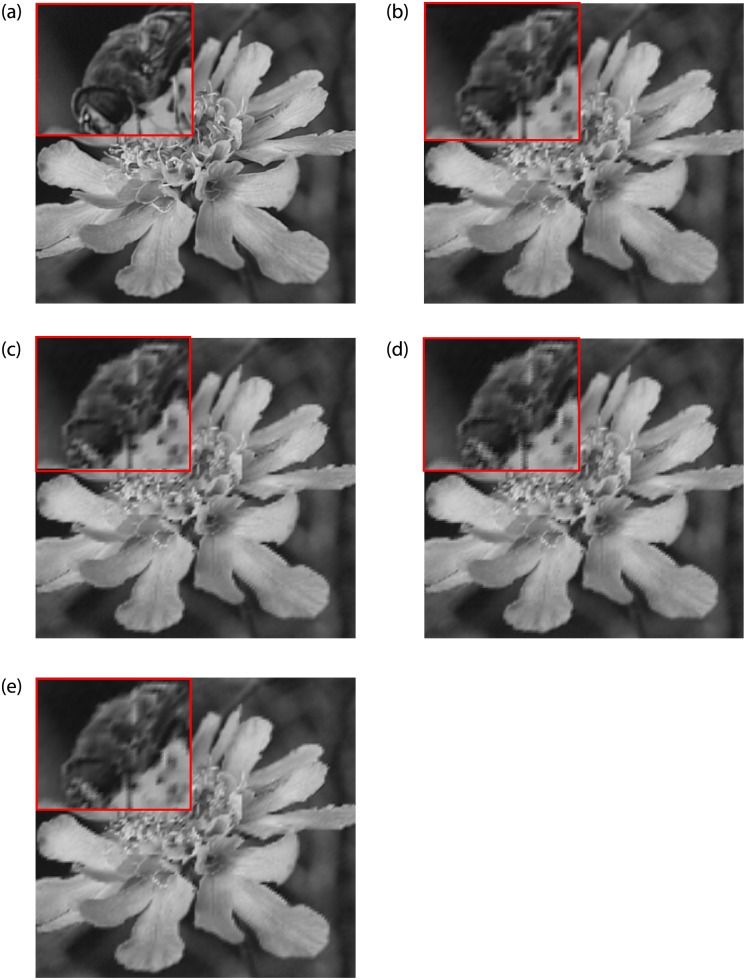
Comparison of Flower images (Interpolation Factor 4). (a) Original image. (b) FCSS. (c) SCSS. (d) FCSV. (e) SCSV.

Figs 12 and 13 show the upscaling images of ‘Peppers’, and ‘Flower’, which are produced by our image interpolation methods with interpolation factor 4. Comparing the visual quality of two standard test images, when the interpolation factor increases, our methods are prone to jagged edges, see Fig 12. Therefore, in terms of processing the images with long edges, our methods are not as good as RBC. But for the images containing texture regions, our methods have advantages, see Fig 13. This result is also reflected in Table 9. For pepper and airplane images, the FSIM values of our methods are lower than RBC. But the difference between the FSIM value of each of our method and RBC is significantly smaller, and even the results of our methods are better.

## 7.Conclusions

In order to overcome the shape defect of the classic Side-Side method and Nielson’s Side-Vertex method, this paper proposes a new class of rational quadratic/linear trigonometric Hermite functions with two shape parameters. Applying them we obtain the new improved first class of Side-Side (FCSS), second class of Side-Side (SCSS), first class of Side-Vertex (FCSV) and second class of Side-Vertex (SCSV) interpolation operators. These improved interpolation methods can be used to construct Coons surface patches and stitch surfaces that meet the following properties:

1. The shape of the Coons surface patch can be flexibly adjusted by altering the values of shape parameters.
2. Always maintain interpolating boundary values and partial derivatives.
3. *C*^1^ continuity.

For the properties that we can adjust the shape of the surface patches using the shape parameters, a center of mass function value control method is proposed. When constructing a triangular patch that requires the center of mass passing through a fixed point, the corresponding parameter values can be calculated by this method. Moreover, FCSS, SCSS, FCSV and SCSV are applied to image interpolation. By adjusting the parameter values, it is possible to obtain images with better quality. The reason is that the Coons surface patches constructed by these proposed methods always satisfy the interpolation of boundary values and partial derivatives. Therefore, these methods ensure that the adjustment of the surface shape is limited to a reasonable range without destroying some of the properties required by the original image. Future work will concentrate on constructing *C*^2^ Coons surfaces over triangular domain with shape parameters.
